# Light-printable epoxy oligomer wrinkle-forming surface for rewritable information storage†

**DOI:** 10.1039/c9ra10569g

**Published:** 2020-01-21

**Authors:** Lin Xu, Umair Azhar, Zizhao Chen, Qingxia Niu, Jian Chen, Xiaohan Zhao, Shuxiang Zhang, Chuanyong Zong

**Affiliations:** Shandong Provincial Key Laboratory of Fluorine Chemistry and Chemical Materials, School of Chemistry and Chemical Engineering, University of Jinan Jinan 250022 P. R. China chm_zhangsx@ujn.edu.cn chm_zongcy@ujn.edu.cn; College of Chemistry, Central China Normal University Wuhan 430079 P. R. China

## Abstract

Smart surfaces with controlled topography show broad and fantastic applications in optics, biology and information science. Herein, we report a simple visible-light-illumination approach to fabricate a smart wrinkle-forming surface with photo-controllable hierarchical surface patterns as well as rewritable high-resolution patterns of information by using an azobenzene-containing epoxy-based oligomer. The epoxy oligomer was synthesized *via* the ring-opening polymerization of bisphenol AF diglycidyl ether (BADFGE) with *p*-aminoazobenzene (AAB) and characterized using FTIR, ^1^H NMR and ^19^F NMR spectroscopies. When the epoxy oligomer film was deposited on an elastic substrate, the formation of surface wrinkles was triggered *via* a circulation of heating/cooling and photo-tailored due to photo-softening together with the release of stress induced by cycles of photoisomerization of azobenzene in the oligomer. The wrinkles in selectively light-exposed regions could be photo-erased within tens of seconds, yielding a different pattern of information. The high-resolution photo-printed images were shown to be rewritable for multiple cycles and legible for over 3 months in dark ambient conditions. The as-formed epoxy oligomer wrinkle-forming surface was found to be inexpensive and its fabrication was easily amenable to scale up, indicating its great potential as ink-free light printable media for rewritable information storage.

## Abstract 2

A novel rapidly responsive media for rewritable information storage comprised of an azobenzene-containing epoxy-based oligomer displaying surface wrinkling was developed.

## Graphical abstract

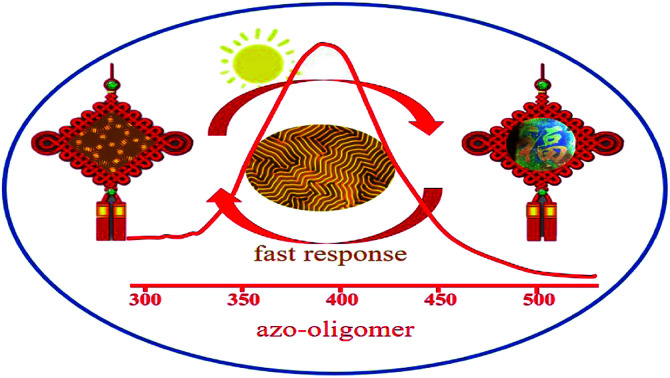

## 1. Introduction

Oligomers with low molecular weights, such as oligomeric proteins, are indispensable for life.^1,2^ In modern scientific research and applications, synthetic oligomer compounds comprising various chemical structures such as biochemical materials, functional dyes, stimulus-responsive components and semiconducting electronic elements play important roles in the fields of biomedicine, optics, organic electronics and information technology.^3^ For instance, the organic semiconducting oligomers including vacuum-deposited P-type oligomers (*e.g.*, acenes, oligothiophenes and selenophene oligomers), N-type oligomers (*e.g.*, quinoid oligomers, perfluorinated oligomers such as rubrene, linear acenes and tetrathiafulvalene derivatives), as well as solution-processed P-type oligomers (*e.g.*, star-shaped oligomers) and N-type oligomers (*e.g.*, naphthalenetetracarboxy diimide derivatives), are extensively utilized as low-cost thin-film transistors displaying excellent processibility.^4^ Thiophene-based conjugated oligomers with 1D to 3D molecular architectures exhibit well-defined molecular structures, better long-range order in the solid state along with excellent processibility as compared to the corresponding high-molecular-weight conjugated polymers, and are essential in the development of high-performance organic solar cells.^5^ Certain synthetic and supramolecular oligomers with helical structures have been extensively used as chirality-sensing systems for applications involving the screening of asymmetric reaction products, pattern recognition of saccharides and enantiomeric excess (ee) measurements, *etc.*^6^ Other oligomers, such as heterocyclic compounds including drugs, pesticides, dyes, and plastics, are also vital in various fields.^7^ Photo-responsive oligomers, for example, azobenzene-containing oligomers, have especially attracted a lot of attention of scientists owing to the special characteristics of these oligomers, namely that their conformations and properties can be photo-controlled in a contact-free and instantaneous manner as well as with high spatiotemporal resolution. Tie *et al.* have described the relationship between the dynamic azobenzene isomerism and the properties of folded azobenzene-containing oligomers, which not only shed light on understanding the interdependence of structure and function in biological systems but also provided a tremendous impetus for the design of synthetic functional systems with folded conformations resembling natural secondary structures.^8^ Yu *et al.* have designed a series of photo-switchable amphiphilic oligo (azobenzene) foldamers and investigated the dynamics of light-induced unfolding processes influenced by chain length and/or different donor and acceptor chromophores.^9^ Ledin *et al.* have synthesized various azo-functionalized star-shaped hybrid nanomaterials with polyhedral oligomeric silsesquioxanes and azobenzene dye as the core and arms, respectively. The branched oligomeric silsesquioxanes with high dye grafting density exhibit high thermal and mechanical stability levels with excellent optical properties and can be used for nanometer-sized photo-switches, optical storage mediums and the fabrication of other optics.^10^ Zha *et al.* have synthesized a new family of azobenzene-functionalized siloxane oligomers with well-ordered molecular arrangements, whose solid-to-liquid transition can be triggered quickly and reversibly by light.^11^ Appiah *et al.* have presented a facile approach to synthesize azobenzene-functionalized linear polyolefin-oligomers with controllable molecular structures, which could form photo-responsive micelles in solution by self-assembling.^12^ Gupta *et al.* have made a series of photo-responsive liquid crystals with star-shaped azobenzene-centered cholesterol-based oligomers as triggers.^13^ Nazmieva *et al.* have developed new nonlinear optical (NLO) polymers with NLO activity based on epoxy-amine oligomers containing bi-chromophore fragments in the side chain.^14^ For information storage applications, azobenzene-containing peptide oligomers,^15^ tolyle-based azobenzene oligomers,^16^ molecular glasses bearing glassy azochromophores,^17–19^ and other low-molecular-weight azobenzene materials^20,21^ have been widely used for holographic data storage based on their photoinduced birefringence and surface-relief-grating formation. However, these are strongly dependent on sophisticated types of equipment and strict operating procedures, and demonstrate poor rewritability. Due to all of the mentioned bottlenecks, it remains a challenge to use azobenzene-based oligomers for rewritable information storage at low cost and high throughput.

Herein, we describe a novel rapid-responsive media for rewritable information storage comprised of the azobenzene-containing epoxy-based oligomer and displaying controllable surface wrinkling. The epoxy oligomer was synthesized *via* the ring-opening polymerization of the bisphenol AF diglycidyl ether (BADFGE) with *p*-aminoazobenzene (AAB) and characterized using FTIR, ^1^H NMR, ^19^F NMR and UV-vis spectroscopies. The formation of wrinkles on the surface of the epoxy-based oligomer film deposited on elastic polydimethylsiloxane (PDMS) substrate was triggered *via* circulation of heating/cooling and these wrinkles could be dynamically tailored or erased using visible-light illumination. The influences of experimental parameters (*e.g.*, wrinkle wavelength and irradiation intensity of the visible light) and the specific mechanism of the evolution of photo-triggered surface wrinkles were systematically investigated. In particular, upon exposing the surface to light through various photomasks, various rewritable patterns of information were constructed conveniently to high resolution within tens of seconds. These photo-printed images with hierarchical surface wrinkled morphologies were rewritable over multiple cycles and remained legible for more than 3 months under ambient conditions in the dark. The low cost and facile ability to scale up the fabrication of the azobenzene-containing epoxy-based oligomer with a wrinkle-forming surface make it a potential candidate as an ink-free light-printable media for rewritable information storage.

## 2. Experimental

### 2.1 Materials

*p*-Aminoazobenzene (AAB, 98%) was purchased from TCI (Shanghai, China). Bisphenol AF diglycidyl ether (BADFGE) was prepared in our laboratory (for detailed information, see ESI Fig. S1†). Polydimethylsiloxane (PDMS) pre-polymer and curing agent (Sylgard 184) were purchased from Dow Corning. All other reagents were commercial analytical products and used directly. Various copper grids, with 200 mesh-sized round holes (R200), 100 mesh-sized square holes (S100), 200 mesh-sized square holes (S200), 300 mesh-sized square holes (S300), and 400 mesh-sized round holes (R400), were purchased from ZKSZ Technology Development, Ltd. (Beijing, China).

### 2.2 Synthesis of azobenzene-containing epoxy-based oligomer (PAFAB)

The azobenzene-containing epoxy-based oligomer (PAFAB) was synthesized by performing solid-state polymerization. Briefly, a mixture of BADGE and AAB with a molar ratio of 1 : 1 was heated at 105 °C with vigorous stirring for 45 h. After cooling, the obtained product was dissolved in tetrahydrofuran (THF), followed by drop-wise addition of the resulting solution into a 2 : 1 vol/vol methanol : H_2_O mixed solvent. The resulting crimson precipitates of PAFAB were filtered and dried in a vacuum oven at 60 °C.

### 2.3 Fabrication of a light-printable PAFAB/PDMS bilayer surface wrinkling system

The PAFAB/PDMS bilayer was prepared by spin-coating the PAFAB/THF solution onto a PDMS substrate with a thickness of ∼2 mm. This substrate had been prepared by mixing the prepolymer/curing agent at a 10 : 1 weight ratio and then performing the curing at 70 °C for 4 h. The PAFAB film thickness was adjusted by varying the spin-coating speed and/or solution concentration. Wrinkles were produced on the PAFAB/PDMS bilayer surface by heating the bilayer at 90 °C for 1 h, followed by gradually cooling it to room temperature. Various samples of wrinkle-forming PAFAB film were modified with different visual information; in each case, selected parts of the film were exposed to light from a halogen lamp (CEL-HXF300) by using a copper grid or photomask conformally covered on the wrinkle-forming sample.

### 2.4 Characterizations

^1^H NMR and ^19^F NMR spectra of samples in CDCl_3_ solvent were acquired by using an Advance III 400 MHz NMR spectrometer (Bruker, Faellanden, Switzerland). Fourier transform infrared (FT-IR) spectra were recorded using a Nicolet Is10 FT-IR spectrophotometer (Thermo Fisher, USA). The molecular weight and dispersity (*Đ*) were analyzed using gel permeation chromatography (Waters 1500) with a column of THF solvent, a high-performance liquid chromatography pump, and a refractive index detector. Differential scanning calorimetry (DSC) measurements were taken using a DSC-Q2000 (TA Instruments Co.). The scanning temperature ranged from 20 to 200 °C, with a heating rate of 10 °C min^−1^ in an N_2_ atmosphere. UV-visible (UV-vis) absorption spectra were acquired by using a TU-1901 spectrophotometer. Optical images were recorded using an inverted Observer A1 microscope (Zeiss, Germany) equipped with a charge-coupled device camera. Atomic force microscopic (AFM) images were obtained using a BenYuan CSPM5500 microscope in tapping mode. The light intensity was measured with an optical power meter (Zhongjiao Aulight, Beijing, China). The surface temperatures of the samples and the corresponding infrared images were characterized by using a Testo 875-1i thermal imager (Testo AG, Germany).

## 3. Results and discussion

**Fig. 1:**
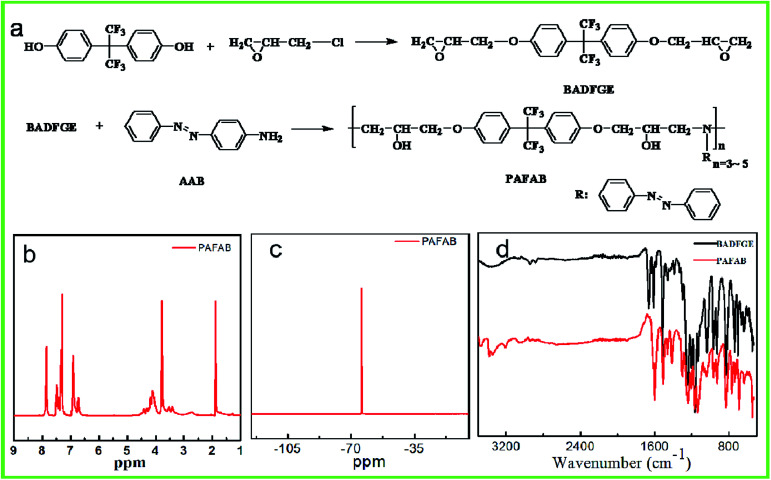
Schematic illustration of the process used to synthesize the epoxy-based oligomer (PAFAB) (a); ^1^H NMR (b) and ^19^F NMR (c) spectra of the PAFAB oligomer; and FTIR analysis of the monomer (BADFGE) and PAFAB oligomer (d).

As shown in Fig. 1a, the photo-responsive azobenzene-containing epoxy-based oligomer was synthesized *via* the ring-opening polymerization of the BADFGE monomer with AAB. All obtained oligomers had a relatively low molecular weight (*i.e.*, *M*_n_ = 2.0 × 10^3^) and a moderate dispersity (*Đ*) (*i.e.*, *M*_w_/*M*_n_ = 1.14) (ESI Fig. S2†). The chemical structures of the epoxy-based oligomer were characterized by acquiring ^1^H NMR, ^19^F NMR and FTIR spectra of them (Fig. 1b–d). As depicted in Fig. 1b, the featured ^1^H NMR peaks at 6.85, 7.38, and 7.85 ppm were due to –CH– groups in the benzene ring (Ar–H) of the oligomer.^22^ The signals at 3.76 and 4.12 ppm were assigned to –CH– and –CH_2_ groups generated from the open loop of the alkylene oxide.^23^ The characteristic peak of –CH_2_– groups connected to oxygen was observed at 1.85 ppm.^23^ Meanwhile, the ^19^F NMR spectrum of the PAFAB oligomer displayed the characteristic absorption peak of the –CF_3_ group at about −64.20 ppm (Fig. 1c).^24^ These results were consistent with the expected chemical structures of the azobenzene-containing epoxy-based oligomer. In addition, the characteristic FTIR bands of the –N– group in the oligomer were observed at 3207 cm^−1^ and 3473 cm^−1^ (Fig. 1d), further indicating the successful synthesis of the azobenzene-containing oligomer.^25^

**Fig. 2:**
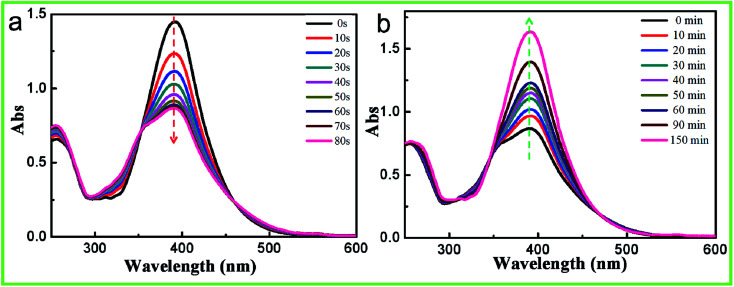
UV-vis absorption spectrum evolution of the oligomer/THF solution (0.018 g L^−1^) upon visible light irradiation (450 nm, 20 mW cm^−2^) (a) and consequent storage in a dark room (b).

Similar to the case for other aminoazobenzenes, the azobenzene chromophore on the side chain of the epoxy-based oligomer yielded *trans* and *cis* absorption bands that overlapped. This overlap was attributed to the electron-donating substituents having caused the π–π* transition to shift to higher wavelengths.^26–28^ Therefore, the UV-vis absorption spectrum of the PAFAB solution/film exhibited a distinctive unimodal curve with a maximum absorption peak at about 390 nm (Fig. 2a and ESI Fig. S3†). Namely, the azobenzene chromophore of PAFAB exhibited a dynamic *trans*/*cis*/*trans* photoisomerization cycle with stable *trans* and metastable *cis* conformations simultaneously converted to an electronically excited state when irradiated with light of the correct wavelength. As shown in Fig. 2a, upon irradiation with 450 nm-wavelength visible light, a gradual decrease in the intensity of absorbance peak of the PAFAB solution was clearly evident. This rapid evolution of the absorbance peak triggered by visible light irradiation was due to the dynamic reversible *trans*–*cis* isomerization of azobenzene molecules. Also, a photo-stationary state was established within 80 s as confirmed by the overlapping features in the spectra recorded at different irradiation times (Fig. 2a). After being subjected to visible light irradiation, the PAFAB solution was preserved in the dark and the maximum absorption band at approximately 390 nm fully recovered to the steady initial state within 150 min, indicating that the *cis* state yielded by light irradiation was completely and reversibly relaxed to the *trans* state (Fig. 2b). This reversible *trans*–*cis* photo-isomerization of azobenzene molecules endowed the azo-containing polymers with various intriguing light-responsive behaviors (*e.g.*, in the current case, photo-softening and photo-triggered stress relaxation), which resulted in the photo-tunable transformation of wrinkles on the surface of the azobenzene-containing epoxy-based oligomer wrinkle-forming film (for a detailed discussion, see the following section).^27–30^

**Fig. 3:**
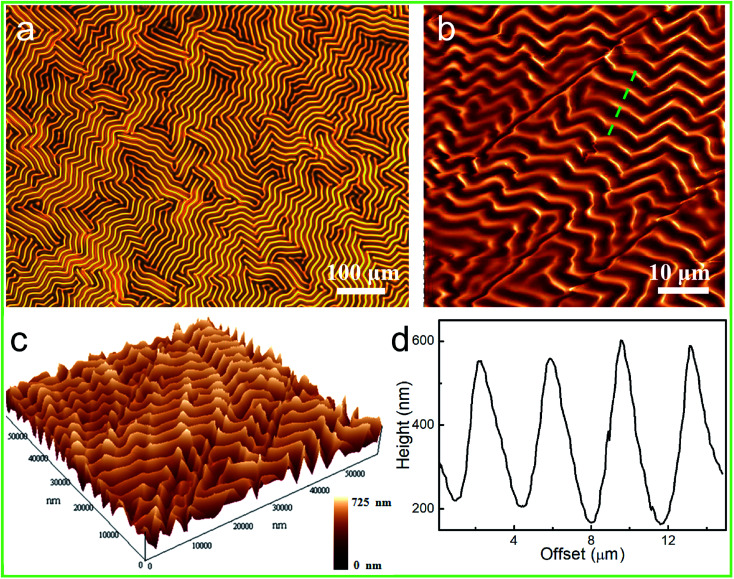
Optical (a), atomic force microscope (AFM) height (b), and three-dimensional AFM (c) images, and the cross-section profile (d) of the heat-generated disordered labyrinth of wrinkles on the PDMS/PAFAB bilayer system.

**Fig. 4:**
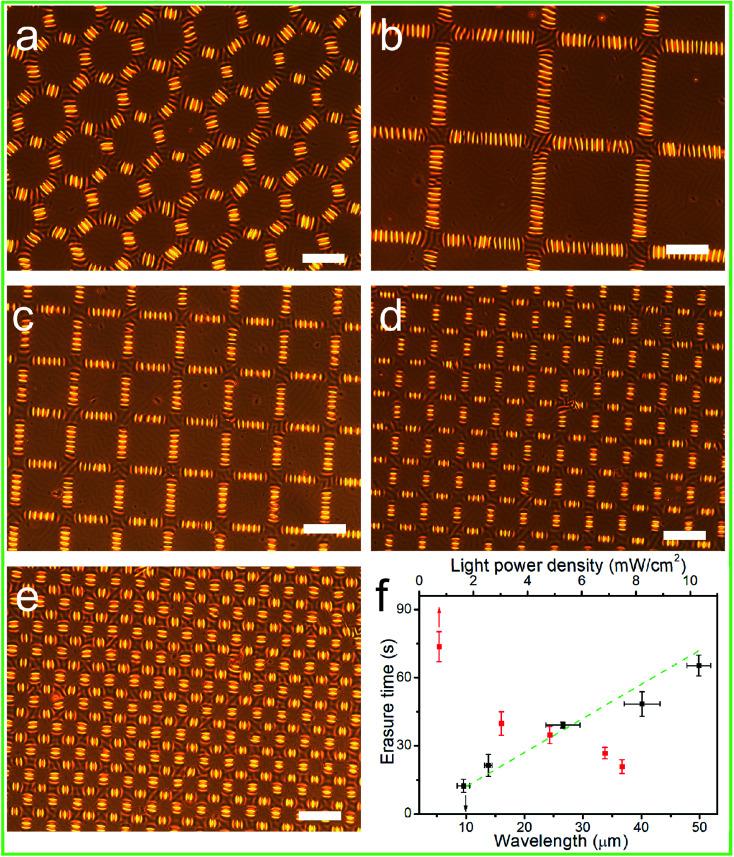
Optical images of the patterns of wrinkles on the PDMS/PAFAB bilayer system after the bilayer system had been selectively irradiated with visible light through copper grids R200 (a), S100 (b), S200 (c), S300 (d), and R400 (e). Frame (f) shows the dependence of the optical erasure time on the light power density *I*_opd_ (*λ* = 21 μm) and on the wrinkle wavelength *λ* (*I*_opd_ = 7.5 mW cm^−2^). Scale bars: 100 μm.

Due to the intrinsic surface instability of the rigid thin films, surface wrinkling took place ubiquitously and spontaneously on these films when they were placed on an elastomeric substrate, as a result of the out-of-plane displacement and once the stimulus-induced compressive strain or stress (*σ*) exceeded the film/substrate system-defined critical wrinkling value (*σ*_c_).^31,32^ As shown in Fig. 3a, the generation of globally homogeneous disordered surface wrinkles was conveniently triggered by the compressive strain introduced *via* the mismatch between the thermal expansion of the film and that of the substrate, after cooling the 90 °C-heated PDMS/PAFAB bilayer system. Wrinkle-forming bilayers are well known to exhibit minimum elastic energy, due to the balance between the bending energy of the stiff film and the energy required to deform the compliant substrate.^33^ And no delamination of the upper PAFAB film was observed after the heating/cooling process. The labyrinth of wrinkles that emerged on the bilayer system displayed a typical sinusoidal profile with an amplitude-to-wavelength ratio of approximately 1 : 20 (Fig. 3b–d), similar to those of the previous reports.^27,29,34,35^ Due to the oligomer bearing the aminoazobenzene group on the side chain, the surface wrinkles of the PDMS/PAFAB bilayer system were photo-responsive. Upon being illuminated with visible light, especially with a selective exposure, the surface wrinkles could be rapidly photo-erased and/or dynamically photo-tailored to yield various hierarchical surface patterns. As demonstrated in Fig. 4a–d, the surface wrinkles in the exposed region had become fully photo-erased, while the initial labyrinth of wrinkles in the unexposed region transformed into highly ordered wrinkles with their orientation perpendicular to the boundary of the exposed region within tens of seconds upon subsequent illumination with visible light. Surface wrinkling, being a type of metastable state, has been well established to display morphologies sensitive to the localized stress field and easily disturbed by external stimuli (*e.g.*, light, mechanical stress, humidity, or solvent).^32,36^ The rapid light-irradiation-triggered *trans*–*cis*–*trans* photoisomerization cycling of the azobenzene chromophore grafted at the amorphous epoxy-based oligomer could induce a photo-softening effect together with a mechanical micro-stress perturbation on the wrinkle-forming film, leading to a residual release of stress in the film and finally a complete erasure of the surface wrinkles in the exposed region.^27–29,37^ Meanwhile, the balance of the localized stress field in the unexposed wrinkle-forming region was disturbed by the boundary effect resulting from the selective exposure to light, leading to a local stress redistribution and consequent dynamic evolution of the orientation of the wrinkles.^31,38,39^ Furthermore, the time required to completely erase the wrinkles in the exposed region, namely the speed of formation of the hierarchical patterns of wrinkles, was strongly dependent on the wrinkle wavelength as well as the light power density (Fig. 4f). As the surface wrinkle wavelength (the film thickness) was increased, increasing residual stress was retained in the wrinkle-forming PAFAB film, which resulted in the longer time needed for the release of compressive stress to lower the film/substrate system-defined critical wrinkling value (*σ*_c_) (ESI Fig. S4†). Conversely, irradiation by visible light with a higher light power density led to a more rapid *trans*–*cis*–*trans* isomerization cycling of the azobenzene groups, which enhanced the release of residual stress in the wrinkle-forming PAFAB film. Note that the time required to print, using visible light, hierarchical patterns of wrinkles on the oligomer-based film/substrate wrinkling system was much shorter than the time it took when using the homologous pseudo-stilbene or aminoazobenzene-containing epoxy-based polymers, which may be attributed to the low-molecular-weight oligomer with less chain entanglement having chain segments more able to move in response to a light trigger (Fig. 4f).^27,28^

**Fig. 5:**
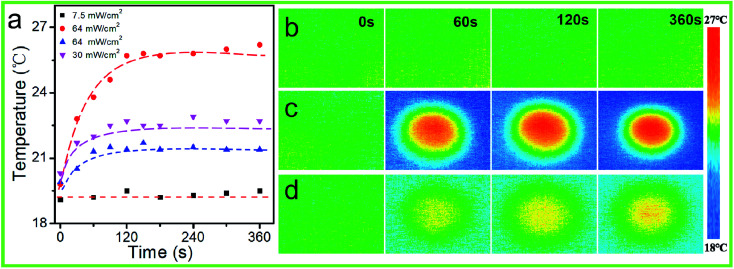
Changes in surface temperature of the wrinkle-forming samples (black square, purple triangle and red circle) and the blank PDMS substrate (blue triangle) under various intensities of visible light irradiation (a); IR camera images revealing the surface temperature of the wrinkle-forming samples (b and c) and blank PDMS substrate (d) during 7.5 mW cm^−2^ (b) and 64 mW cm^−2^ (c and d) light irradiation.

Owing to the low glass transition temperature (*T*_g_ ≈ 56 °C) of the PAFAB oligomer (Fig. S5†), the photo-thermal effect on the wrinkle-forming samples surfaces was systematically investigated. As shown in Fig. 5, the temperature change on the surface of the sample with different light irradiation intensities was *in situ* monitored using a Testo 875-1i thermal imager. When irradiated with high-intensity light (*e.g.*, 64 mW cm^−2^), the surface temperature increased slightly within 120 s, with a temperature change of only about 6 °C (Fig. 5a and c), which was similar to the values in previous reports.^40–42^ And the surface temperature increase on the corresponding blank PDMS substrate was about 2 °C during light irradiation (64 mW cm^−2^) (Fig. 5a and d). But when irradiated with low-intensity light (*i.e.*, 7.5 mW cm^−2^), which we used for photo-regulating the surface wrinkles of the PDMS/PAFAB system, no obvious surface temperature change was observed during the light irradiation (Fig. 5a and b). Namely, no photothermal-induced segmental motion occurred in the wrinkle-forming oligomer film during the photo-triggered evolution of surface wrinkles with the sample surface temperature well below the *T*_g_ of the PAFAB oligomer. Overall, the results further confirmed that the photo-controlled evolution of the surface wrinkles of the PDMS/PAFAB system was due to the photo-softening effect together with the mechanical microstress perturbation-triggered compressive stress release induced by the *trans*–*cis*–*trans* isomerization cycles of azobenzene in the wrinkle-forming oligomer film. It is worth noting that compared with the blank PDMS substrate, the wrinkle-forming azobenzene-containing film exhibited a greater surface temperature increase during light irradiation, due to the light absorption of the azobenzene-containing epoxy-based oligomer (Fig. 5a, c and d, and 2). This considerable photothermal conversion ability of the azobenzene-containing materials could be used to fabricate diverse on-demand azo-based hierarchical patterns of wrinkles with high aspect ratios with the application of high-intensity white-light-illumination (*e.g.*, *I*_opd_ = 1.8 W cm^−2^).^30^

**Fig. 6:**
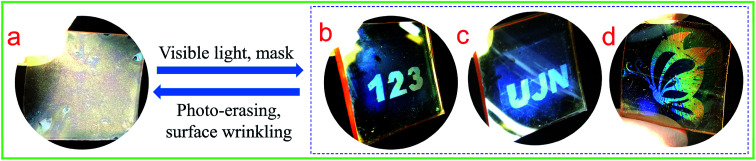
Application of the PDMS/PAFAB wrinkling system for rewritable information storage as a result of selective exposure to visible light: as-fabricated wrinkle-forming surface (a); various patterns of wrinkles obtained by selective exposure *via* different photomasks (b–d).

The epoxy-based PDMS/PAFAB wrinkling system could be used as rapidly responsive and easy-to-use media for large-area information storage based on the excellent photo-responsiveness of the surface wrinkles of the azo-oligomer film combined with light as the external stimulus, which is conveniently spatiotemporally controlled and involves a contact-free path. As presented in Fig. 6, by means of the simple selective visible light exposure, various visualized patterns of information were clearly printed using light on the PAFAB films due to the unexposed wrinkle-forming regions exhibiting a lower transmittance and stronger scattering. Amazingly, the light-printed high-resolution patterns of information demonstrated excellent stability in the dark, being legible for more than 3 months under ambient conditions, and were rewritable over multiple cycles (Fig. 6b–d). Moreover, the as-prepared azobenzene-containing epoxy oligomer wrinkle-forming surface was found to be inexpensive and easily amenable to large-scale fabrication, and hence may find more advanced potential applications in sensors, soft photonics, microfluidics and so on.^36^

## 4. Conclusion

In summary, we have reported a facile and universal strategy to fabricate a photo-responsive wrinkle-forming surface for rewritable information storage based on the synthesized azobenzene-containing epoxy-based oligomer. The surface wrinkles on the oligomer film were photo-regulated or photo-erased owing to the photo-softening effect and the stress release triggered by the photo-isomerization cycles of the azobenzene groups. Various hierarchical surface wrinkles, as well as photo-rewritable high-resolution information micropatterns on the epoxy-based oligomer films, were conveniently fabricated upon their being selectively exposed to visible light. Moreover, as compared to the wrinkling system with high-molecular-weight epoxy-based azo-polymers as the stiff film, the surface wrinkles of the oligomer-based film/substrate system exhibited more rapid photo-responsiveness. As demonstrated in this research, this simple method to construct a photo-responsive wrinkle-forming surface for information storage can be conveniently extended to other functional oligomer systems with unprecedented advantages in patterning controllability and universality.

## Conflicts of interest

There are no conflicts to declare.

## Supplementary Material

RA-010-C9RA10569G-s001
