# Supplementary material for: Light-printable epoxy oligomer wrinkle-forming surface for rewritable information storage
Source: RSC Adv. 2020 Jan 21;10(6):3416–23. doi: 10.1039/c9ra10569g (PMC9048581; doi:10.1039/c9ra10569g)
Supplement: RA-010-C9RA10569G-s001 [file RA-010-C9RA10569G-s001.pdf]

Electronic Supplementary Material (ESI) for RSC Advances.
This journal is © The Royal Society of Chemistry 2020

Supporting Information

Light-Printable Epoxy Oligomer Wrinkling Surface for Rewritable Information Storage

Lin Xu,[†] Umair Azhar,[†] Zizhao Chen,[‡] Qingxia Niu,[†] Jian Chen,[†] Xiaohan Zhao,[†] Shuxiang Zhang,[*,†] and Chuanyong Zong[*,†]

[†]Shandong Provincial Key Laboratory of Fluorine Chemistry and Chemical Materials, School of Chemistry and Chemical Engineering, University of Jinan, Jinan 250022, P. R. China.

[‡]College of Chemistry, Central China Normal University, Wuhan 430079, P. R. China

Corresponding Authors

***E-mail:** chm_zhangsx@ujn.edu.cn

***E-mail:** chm_zongcy@ujn.edu.cn

Materials. Hexafluorobisphenol A (6FBPA, 98%) was purchased from Sigma-Aldrich. Epoxy chloropropane, tetrabutylammonium bromide, N,N-dimethylformamide (DMF) and tetrahydrofuran were purchased from Sinopharm Chemical Reagent Co. Ltd. (Shanghai, China). All other reagents were commercial analytical products and used directly.

Synthesis of bisphenol AF diglycidyl ether (BADFGE). The BADFGE was prepared in our laboratory according to a previously reported literature.[1] Briefly, hexafluorobisphenol A (5 g, 0.015 mol), epoxy chloropropane (5.5 g, 0.059 mol) and tetrabutylammonium bromide (0.05g, 0.155 mmol) were added into a round-bottom flask and stirred for 30 min at room temperature. Then, the NaOH solution (8.3 g, 10 mol/L) was added drop-wise to the mixture and stirred for another 12 h. The resulting mixture was washed with water and extracted by diethyl ether, after removing the solvent, the light tan syrupy product (BADFGE) was obtained.

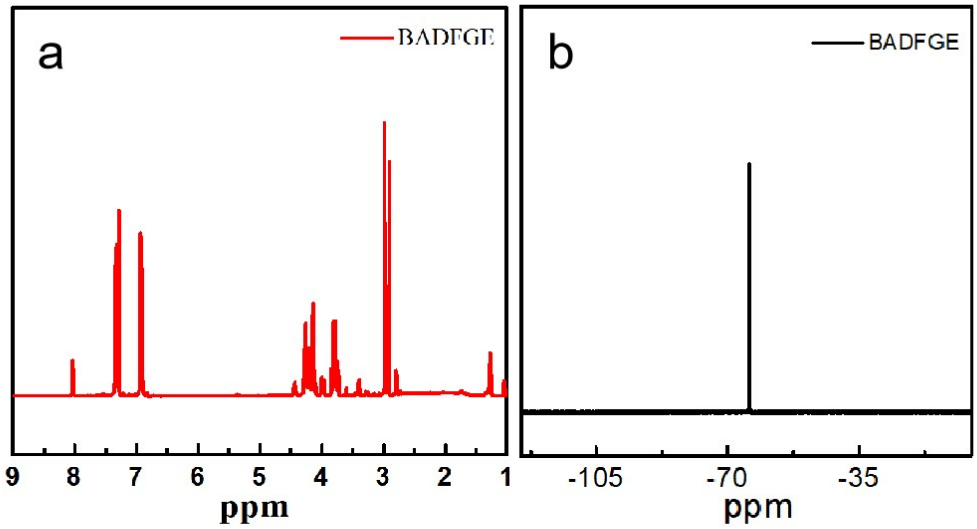

Fig. S1 ^{1}H NMR (a) and ^{19}F NMR (b) spectra of the monomer (BADFGE).

The characteristic peaks at 6.93 and 7.33 ppm were belonged to -CH- groups in the benzene ring. The feature peaks at 3.72-4.33 ppm were assigned to -CH- and $-CH_2$ groups in the epoxy ring. The signals of the methylene groups connecting to the epoxy ring emerged at 1.27 ppm (Fig. S1a).[2] Meanwhile, the ^{19}F NMR spectrum of the BADFGE monomer displayed the characteristic absorption peak of $-CF_3$ group at around -64.25 ppm (Fig. S1b).[3]

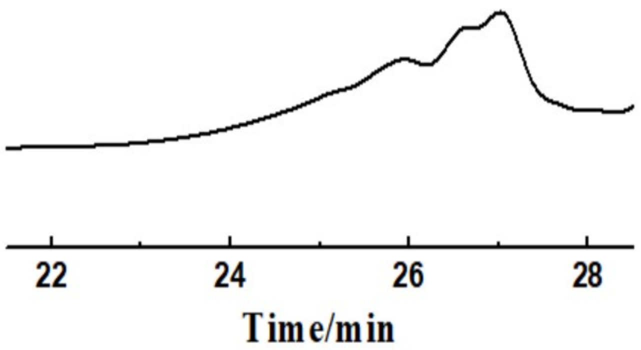

Fig. S2 GPC traces of the epoxy-based oligomer.

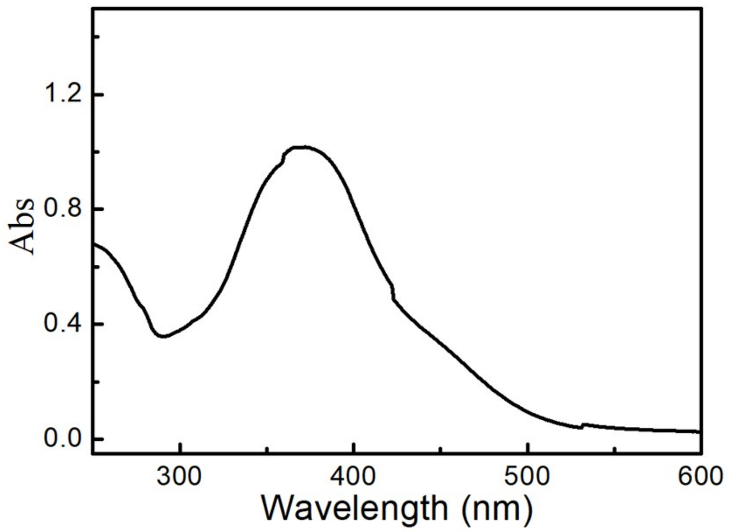

Fig. S3 UV-vis absorption spectra of the oligomer film deposited on the polydimethylsiloxane (PDMS) substrate.

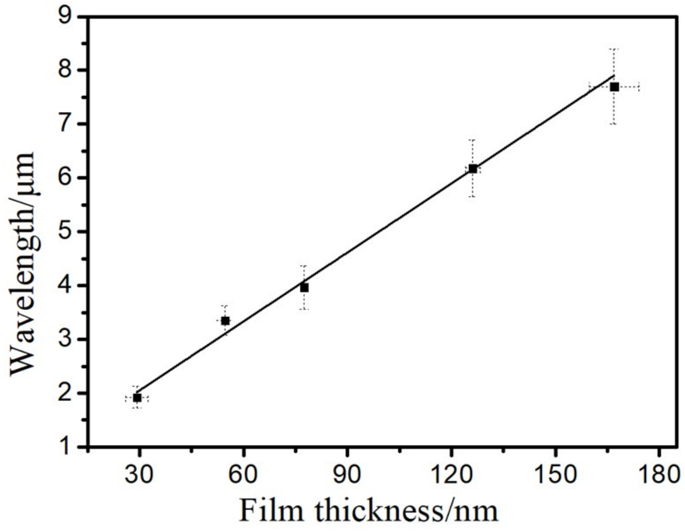

Fig. S4 Dependence of the wrinkle wavelength of the PDMS/PAFAB bilayer system on the PAFAB film thickness.

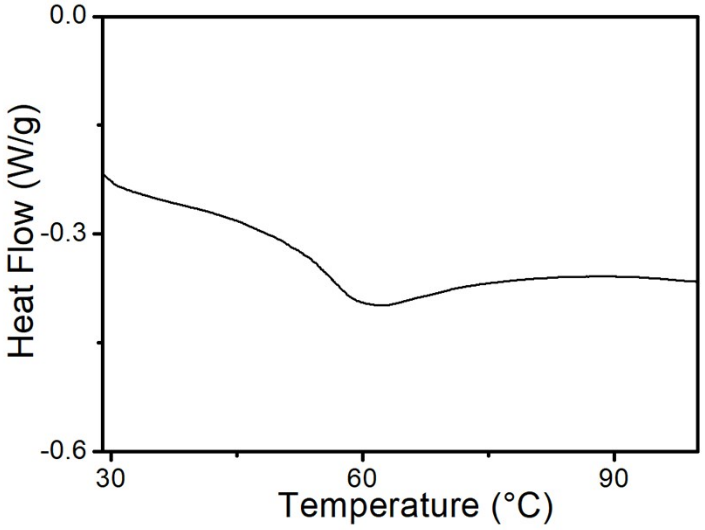

Fig. S5 DSC traces of the epoxy-based oligomer.

Notes and references

[1] Zhou Y, Jiang C, Zhang Y, et al. Structural optimization and biological evaluation of substituted bisphenol a derivatives as *β*-Amyloid peptide aggregation inhibitors[J]. Journal of medicinal chemistry, 2010, 53(15): 5449-5466.

[2] Garcia F G, Soares B G. Determination of the epoxide equivalent weight of epoxy resins based on diglycidyl ether of bisphenol A (DGEBA) by proton nuclear magnetic resonance[J]. Polymer Testing, 2003, 22(1): 51-56.

[3] Borkar S, Jankova K, Siesler H W, et al. New highly fluorinated styrene-based materials with low surface energy prepared by ATRP[J]. Macromolecules, 2004, 37(3): 788-794.
